# The need for action by evaluators and decision makers in Europe to ensure safe use of medical software

**DOI:** 10.3389/fmedt.2022.1063622

**Published:** 2022-11-29

**Authors:** Mattias Kyhlstedt

**Affiliations:** Synergus RWE, Åkersberga, Sweden

**Keywords:** regulation, digital health, health technology assessment, medical software, MDR, MDD

## Abstract

Digital Health Solutions (DHS) approved under the Medical Device Directive (MDD) in the European Union may be used until May 27, 2025. The regulation provides appropriate requirements for the products but lack the evaluation by an external independent organization. For many DHS, the company can make a self-certification that the requirements have been fulfilled. As demonstrated in the evaluation of smartphone-based apps for skin cancer risk assessment such products may expose the public to undue risks. The new Medical Device Regulation provides adequate control of DHS through evaluation of independent organization prior to allowing the product on the market. HTA-evaluators and those who make decisions regarding the use of DHS need to understand the associated risks with the use of products approved according to the MDD and ensure appropriate risk mitigations to ensure that the public is not exposed to undue risk. This perspective aims to inform decisionmakers about the risks associated with the delayed requirement to transition to the new MDR regulation. There is a gap in the current guidance regarding the evolving use of machine learning and artificial intelligence. With the evolving use of DHS, it is important that industry, regulators and HTA evaluators work jointly to establish the safe and effective use of DHS.

## Introduction

The proliferation of digital health solutions (DHS) entering the market has led to increased concern about both quality control and user safety. A recent systematic review of diagnostic accuracy studies of smartphone applications for skin cancer risk assessment by Freeman et al. concluded: “*The current regulatory process for awarding the CE marking for algorithm based apps does not provide adequate protection to the public*.” (1). The limitations of the MDD for class I devices in ensuring the safety of the public were thus made clear.

Originally released in 1993, limitations in the MDD have previously been pointed out, including outdated regulations, lack of consistency, focus on approval and not post-market performance, insufficient scrutiny of notified bodies (2), and the lack of a Software as Medical Device (SaMD) (3) classification, a class of products arguably not foreseen when the regulation was developed. These criticisms led to the establishment of a European Commission medical device working group in 2002 that included several stakeholders (4). The group's work resulted in the revised Medical Device Regulation (MDR), which was enacted in 2017. The MDR addressed several important aspects, in particular the classification of SaMD and the improved selection and oversight of the notified bodies. Whilst the new MDR legislation was initially set to replace MDD on May 26, 2020, a subsequent corrigendum (5) now permits products to remain on the market in their existing form until May 27, 2025. Analysis of the impact of this delayed implementation on how SaMD may operate in the meantime is required, not least from a safety perspective.

The transition from MDD to MDR is of particular importance for software-only products, as there are significant changes in MDR relating to the risk classification. In a study by the National Institute for Public Health and the Environment in the Netherlands (RIVM) a clear majority of products in class I were upgraded to a higher risk class. Indeed, many DHS may currently lead to detrimental health impacts (e.g., by not correctly identifying a skin cancer) through inadequate or incomplete control of the product. As such, any decision to approve use of such technology should include a careful evaluation of the quality, reliability, and validation of health risk assessment products, which are increasingly smartphone-based and self-administered.

## Objective

The objective of this study is to assess if the risks identified in the review of algorithm-driven smartphone-based applications (1) would obtain adequate regulatory control under the new MDR regulation. This study furthermore provides a summary of the main differences between the MDD and MDR and suggests ways to mitigate potential safety issues until the MDR is in full force.

## Methodology

A comparative review of the MDD and MDR legislative frameworks, associated guidance, and standards for the SaMD approval process in the EU was conducted. The review evaluates the difference in regulatory classification and how this impacts the measures to ensure a safe placement of the product on the market.

Eventual limitations in the legislative requirements or the external evaluator's role and/or function were also assessed.

## Results

### MDD vs. MDR regulation

#### Understanding the legislative framework

The MDD requires that DHS products satisfy its Essential Requirements (ER); the corresponding stipulation in the MDR is that they satisfy its General Safety and Performance Requirements (GSPR). The ER include a high-level requirement of all aspects required to ensure a safe medical device or SaMD, including usability software development, across all risk classes of medical devices or SaMD.

For devices including software, the sole requirement stated in the MDD is (6):

*12.1 For devices which incorporate software or which are medical software in themselves, the software must be validated according to the state of the art taking into account the principles of development lifecycle, risk management, validation and verification.*

This requirement arguably needs to be put in context to be clarified. The MDD is based on the availability of harmonized standards that define how the ER best are fulfilled (6):

*Member States shall presume compliance with the essential requirements referred to in Article 3 in respect of devices which are in conformity with the relevant national standards adopted pursuant to the harmonized standards the references of which have been publishes in the Official Journal of the European Communities; Member States shall publish the references of such national standards*.

In the case of software development, the IEC-62304 harmonized standard is applied, providing a well-designed methodological framework for how software should be developed and validated to ensure a safe product. The international standards are developed with methodological experts from industry and legislators in a comprehensive manner. The MDD does not require that this standard be used, but it requires that state of the art be used. If a company can justify another method that represents the state of the art, then they may use that approach instead; it is difficult to pursue such attempt in practice, however. As outlined in Table 2, the documentation for the software development will not be reviewed by anyone if the product belongs to class I in the MDD, whereas both the process and the result will be reviewed for products in class IIa, IIb and III MDR.

#### Risk classification of products leads to better safety control measures

The class of the SaMD determines the measures applied by Notified Bodies to ensure the safety of the products being placed on the market. Some low-risk-class SaMD will remain class I in the MDR as well, such as prevention-based applications e.g., cardio training apps offering workout recommendations (7). How class I SaMD under the MDD might be re-classified as IIa, IIb or III under the MDR requires a more complex analysis of the application of measures, although Table 2 provides a simplified explanation.

#### Comparing the risk classification for DHS

The classification of medical devices, including SaMD, is based on the potential risk associated with the use of the device in relation to the vulnerability of the human body (8). Based on the present MDD (and corresponding guidance) for classification of DHS (9) SaMD according to the MDD is classified as class I in most scenarios, whereas according to the MDR and corresponding guidance most current devices will be classified as class IIa, IIb or even III (10). The 2018 RIVM report (11) compared the classification between the MDD and MDR across 56 categories of SaMD, and found that 73%(24 of 33) of MDD class I devices will be re-classified as Class IIa or higher in the MDR; an additionally, 12% (2 of 16) of MDD Class IIa SaMD devices would be re-classified upwards in the MDR. These re-classifications will lead to different conformity assessment routes, representing different level of safety assurance controls.

#### The role of the notified body

The MDD and MDR rely on commercial notified bodies designated by the competent authorities. This system is not without its flaws however. The BMJ and other publications (12) (13–18) used covert methods involving a fake hip implant to illustrate how the intended control process of the Notified Bodies could be circumvented to get a sub-standard product approved. The UK health secretary, Jeremy Hunt, subsequently pledged to stop this “worrying and completely unacceptable weakness in the regulatory system” (19). This promised scrutiny led to a reduction in MDD-related Notified Bodies from 75 to 58 over several years.

Recognizing the limitations in designating Notified Bodies under the MDD, the accreditation process has been reworked in the MDR. As of February 2020, there were only 11 notified bodies who had been authorized according to the new regulation, the first of which was accredited in January 2019 (BSI). Even though it is not possible to fully understand the effect of the revision, the requirements have increased significantly.

#### Clinical data and literature reviews in the approval process of SaMD

The MDD and MDR require companies to make systematic evaluation of the evidence supporting the utility, performance, and safety of their product, both during initial release of the product and as part of their continued surveillance of the safe use of their product.

Systematic reviews of categories of products, outcomes of relevance and outcomes in standard of care are essential to both developers and evaluators in identifying benefits and risks, and if these are appropriate in relation to the current standard of care. The review of smartphone apps for skin cancer (1) provides an excellent example of how such research can constitute a baseline for future evaluation of technologies in a given product category.

In addition to the review of clinical evidence, §23 in the MDR also outlines the opportunity to develop common standards and specifications for specific product categories (20).

#### Absence of harmonized standards for SaMD in the MDR

There are currently no standards harmonized with the MDR, which creates a high degree of uncertainty for SaMD developers about how they fulfil the requirements in the GSPR, which are equally non-specific as in the MDD. The standards that are harmonized under the MDD are the same as those planned for harmonization with the MDR, although according to current plans this will not be completed until May 2024.

The Medical Device Coordination Group (MDCG) issued guidance for cybersecurity December 2019 (21) that on how these requirements should be addressed. It also included a list of 16 standards for various software development aspects including risk management, usability, network safety, and software life cycle development, although they pointed out that these were solely for informative purposes as they were not harmonized. It is therefore unclear what role these may have in ensuring fulfilment of the GSPR.

#### Guidance and standards regarding artificial intelligence (AI) and machine learning (ML)

There are currently no standards or guidance regarding the design and use of AI/ML. The MDCG has not published any current plans to do so on their publicly available activities list. There is, however, significant pressure from many stakeholders to establish such standards and guidance to ensure the safe use of AI/ML.

### A regulatory perspective

The major quality and performance issues identified in Freeman et al. were cross-tabulated with the relevant sections of the current MDD regulations in Table 1, including an assessment of the adequacy of the MDD for the specific issue (1). The analysis suggest that the quality and performance issues were not likely to be related to the applicable requirements for such products in the regulation but rather to methods applied to ensure compliance under the MDD regulation. With the new MDR regulation, with a revised classification and increased control measures of notified bodies and more stringent requirements for clinical evidence it is unlikely that flaws such as those reported in Freeman et al. would be placed on the market (1).

**Table 1:** Comparison of DHS quality issues identified in the Freeman et al. (1) systematic review vs. regulatory requirements in the MDD.

MDD section	Requirement in MDD	Conclusion from article	Assessment of regulation
Annex I, §1	The devices must be designed and manufactured in such a way that, when used under the conditions and for the purposes intended, they will not compromise the clinical condition or the safety of patients, or the safety and health of users or, where applicable, other persons, provided that any risks which may be associated with their intended use constitute acceptable risks when weighed against the benefits to the patient and are compatible with a high level of protection of health and safety. This shall include: — reducing, as far as possible, the risk of use error due to the ergonomic features of the device and the environment in which the device is intended to be used (design for patient safety), and — consideration of the technical knowledge, experience, education and training and where applicable the medical and physical conditions of intended users (design for lay, professional, disabled or other users).	“Smartphone algorithm-based apps for skin cancer all include disclaimers that the results should only be used as a guide and cannot replace healthcare advice. Therefore, these apps attempt to evade any responsibility for negative outcomes experienced by users. Nevertheless, our review found poor and variable performance of algorithm-based smartphone apps, which indicates that these apps have not yet shown sufficient promise to recommend their use”.	The requirements in the directive should ensure the safe use of products under normal use.
Annex 1, §2	The solutions adopted by the manufacturer for the design and construction of the devices must conform to safety principles, taking account of the generally acknowledged state of the art. In selecting the most appropriate solutions, the manufacturer must apply the following principles in the following order: — eliminate or reduce risks as far as possible (inherently safe design and construction), — where appropriate take adequate protection measures including alarms if necessary, in relation to risks that cannot be eliminated, — inform users of the residual risks due to any shortcomings of the protection measures adopted. supplemented by the harmonized standard for risk management ISO-14971.	“Concern exists about the impact of false reassurances that algorithm-based apps could give users with potentially malignant skin lesions, especially if they are dissuaded from seeking healthcare advice. The current CE marking assessment processes are inadequate for protecting the public against the risks created by using smartphone diagnostic or risk stratification apps”.	There are several aspects related to the risk identified in the article should be addressed by the risk management process of the company. This also includes foreseeable misuse which includes the consideration that the user may use it for another purpose than the limited scope defined in the intended use.
Annex X, §1.1	As a general rule, confirmation of conformity with the requirements concerning the characteristics and performances referred to in Sections 1 and 3 of Annex I, under the normal conditions of use of the device, and the evaluation of the side-effects and of the acceptability of the benefit/risk ratio referred to in Section 6 of Annex I, must be based on clinical data. The evaluation of this data, hereinafter referred to as “clinical evaluation”, where appropriate taking account of any relevant harmonized standards, must follow a defined and methodologically sound procedure based on supplemented by the harmonized standard for clinical trials ISO-14155	Assessment of the validity and applicability of the evidence using QUADAS-2 demonstrated high risk about applicability and in some instances also a high risk of bias.	The studies should follow high methodological standard to ensure the quality of the results.
Annex X, §2.1	The objectives of clinical investigation are: — to verify that, under normal conditions of use, the performance of the devices conform to those referred to in Section 3 of Annex I, and — to determine any undesirable side-effects, under normal conditions of use, and assess whether they constitute risks when weighed against the intended performance of the device.	Firstly, smartphone apps are typically targeted at the general population with a relatively low prevalence of malignant lesions and a wide range of different skin conditions. Studies failed to recruit samples representative of this population.	The regulation requires that it should demonstrate during the clinical trial that it operates as intended during normal conditions and as such should include the appropriate representation of the population.

When DHS products are denoted as class I in the MDD, responsibility for the interpretation of the legislation and harmonized standards lies entirely with the developing company without any involvement of external governance. In high-risk classes, the company and the product will undergo review by a Notified Body. The lack of adequate risk classification for SaMD in MDD and consequent lack of external evaluation is the probable cause for the identified issues in reference (1).

**Table 2:** Comparing measures to ensure safe use of the products on the market.

Category of measure to ensure safe product	Class I – MDD	Class IIa– MDR	Class IIb – MDR	Class III - MDR
External audit by notified body to ensure that the fulfilment of requirements to quality system including the development of software	None	- Prior to allowing the product on the market - Annual - At least one unannounced audit every 5 years.	- Same as IIa	- Same as IIa - At least one unannounced audit every 3 years
Review of technical documentation of product by notified body, including basic clinical data.	None	- Sample review per category of device for initial approval.	- Same as IIa and: - Follow up reviews based on sampling plan.	- Review of every new product.
Review of Safety and clinical performance by notified body (more extensive clinical review)	None	None	None	- Prior to allowing product on the market. - At least annually
Review of post-market follow up	None	- Review based on sampling plan.	- Review based on sampling plan	- Review annually

## Discussion

With the current limitations of how SaMD are regulated under the MDD, it is imperative that stakeholders understand the risks associated with acquiring and using such products. Companies producing class I SaMD products under the MDD can at their discretion claim that both they and their product fulfil the ER, without the need for any external evaluation. As illustrated in the example with of apps for skin cancer (1), such products aim to directly or indirectly influence the diagnosis of cancer, and are currently being placed on the market with inferior performance to clinical standards. This is a potentially major risk public health.

### Suggested actions for developers of SaMD

Developers should ensure they have a good understanding of the regulatory requirements that relate to all aspects of the MDD and MDR frameworks to ensure the ability to provide safe products to the patients. In the absence of harmonized standards under the MDR, the advice is to start implementing the standards that currently are harmonized under the MDD until more clarity has been established for the former.

### Suggested actions for HTA evaluators and decision makers in the use of SaMD

Considering the limitations of the MDD for SaMD and the ability to place such products on the market until May 2025, it is essential that HTA evaluators and decision makers carefully evaluate the associated risks with using such devices.

For Class I SaMD under the MDD, it should be recognized that there is no independent evaluation of the development of the product nor evaluation of the technical documentation and/or clinical data.

This could be mitigated by only approving the use of SaMD that can demonstrate fulfilment of the MDR requirements, or by requiring competent review that the requirements of the MDD have in fact been fulfilled. Companies would argue that this is prohibitive due to the limitations of Notified Bodies in qualifying them according to the MDR, but from a public health perspective is it reasonable to accept the use of SaMD without such assurances?

## Strengths and limitations

This work builds upon the strengths of Freeman et al. (1) in illustrating the consequences of the challenges related to current regulatory situation for SaMD, and adding knowledge of regulatory processes from industry and agencies.

The stakeholders addressed in this study – namely Notified Bodies and regulatory agencies – have not been consulted regarding their view on the conclusions of this article, which may be considered a limitation.

## Conclusion

Active engagement with decision makers and evaluators to create an understanding of the risk at hand of using devices approved under the MDD class I is essential. With the evolving use of DHS in healthcare it is important for industry, regulators, and HTA-evaluators to jointly work together for the safe and effective use. This is in particular important in the growing field of applications using machine learning or artificial intelligence.

## Data Availability

The original contributions presented in the study are included in the article/Supplementary Material, further inquiries can be directed to the corresponding author/s.
